# Ultrasound Monitoring of Umbilical Catheters in the Neonatal Intensive Care Unit—A Prospective Observational Study

**DOI:** 10.3389/fped.2021.665214

**Published:** 2021-04-30

**Authors:** Alina Sobczak, Aleksandra Dudzik, Piotr Kruczek, Przemko Kwinta

**Affiliations:** Neonatal Intensive Care Unit, Department of Pediatrics, Jagiellonian University Medical College, Kraków, Poland

**Keywords:** umbilical catheters, catheter-related thrombosis, bedside monitoring, neonatal intensive care unit, prematurity

## Abstract

**Introduction:** Umbilical catheterization provides a quick yet demanding central line that can lead to complications seen nowhere else. The aim of our study was to determine whether the repeated ultrasound scanning can influence the catheterization time, prevent some of the catheter-related complications, support the decision-making process and allow prolonged catheterization in patients without an alternative central access route.

**Methods:** A prospective observational study was performed in a tertiary neonatal intensive care unit. A total of 129 patients and 194 umbilical catheters (119 venous and 75 arterial) were analyzed with a total of 954 scans. Ultrasound screening consisted of 1) assessing the catheter tip, location, movability, and surface and 2) analyzing the catheter trajectory. The outcome variables were defined as 1) catheter dislocation and 2) associated thrombosis.

**Results:** Dislocation of catheter throughout the whole catheterization period was observed in 68% (81/119) of UVCs and 23% (17/75) of UACs. Thrombotic complications were observed in 34.5% (41/119) of UVCs and 12% (9/75) of UACs. 1/3 of UAC-associated thrombi were visible only after catheter removal. 51% (61/119) of UVC patients and 8% (6/75) of UAC patients made a clinical decision regarding the obtained catheter image.

**Conclusion:** Bedside ultrasound imaging of catheters supports the decision-making process related to the catheterization duration, shortening the time if abnormalities are detected and allowing a safer prolonged UC stay when an alternative central line cannot be inserted.

## Introduction

Umbilical catheters (UCs) are a unique form of central arterial and venous access that can be applied only within the first hours of life. They enable an easy, quick, and painless catheterization route, sparing the other main vessels of the smallest patients for the future. Obtaining vascular access is a condition sine qua non of intensive care enabling drug and fluid administration, parenteral nutrition, continuous blood pressure monitoring, blood sampling, and more; however, acquiring such access when the patient's vessels are extremely small is obviously a challenge. Introducing the usage of dissected umbilical vessels that are easily visible and gaping in the freshly cut umbilical stump causes a rapid increase in the neonatal survival rate; nevertheless, we should not ignore the possible complications they might generate.

To date, there are no clear recommendations regarding the monitoring of umbilical catheters. Since 1946 (1), when umbilical catheterization was first applied for exchange transfusion by Diamond (2), chest radiography has been the technique of choice for assessing the position of a catheter; however, due to radiation concerns, it is usually performed only once after catheter insertion. At the same time, neonatal point-of-care ultrasound plays a growing role in intensive care—lately a Working Group of the European Society of Pediatric and Neonatal Intensive Care provided an evidence-based clinical guideline for the use of point-of care ultrasound in critically ill neonates and children (February 2020) (3) recommending its use by clinicians; however, the role of umbilical catheter monitoring remains unclear.

The aim of our study was to determine the role of systematic ultrasound screening in decision-making in the neonatal intensive care unit. We hypothesized that repeated ultrasound scanning can influence the catheterization time, prevent catheter-related complications and allow prolonged catheterization in patients without an alternative central access route.

## Methods

### Study Design

A prospective observational study was conducted in a tertiary neonatal intensive care unit (NICU) in the Department of Pediatrics, Jagiellonian University between February 1, 2016 and April 30, 2019. The participants were consecutively recruited NICU patients of any weight or gestational age who had a UC inserted, and informed consent was obtained from the parents.

### Ultrasound Imaging

Ultrasound imaging was performed directly after insertion, within the first 24 h and then every 1–2 days or more often if the patient was unstable. Bedside examinations were performed using a Philips HD11 ultrasound system with a linear probe either by a certified neonatologist or a trained pediatric resident; all examinations were recorded and verified by a second examiner.

**Figure 1:**
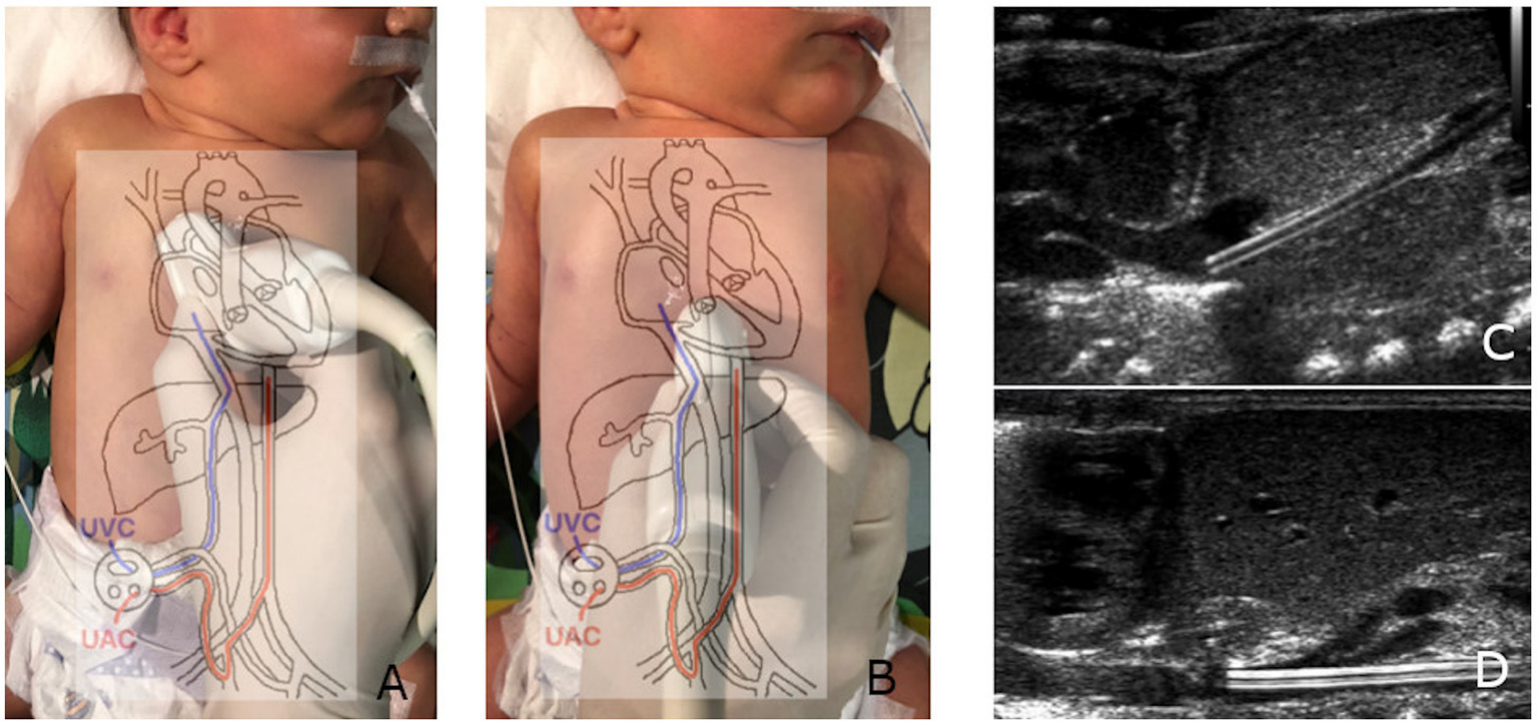
Proper UVC **(A)** and UAC **(B)** imaging and positioning. The UVC leaving the ductus venosus **(C)**; the UAC, crossing the celiac trunk and superior mesenteric artery **(D)**.

The catheters were analyzed from a frontal thoracic view; to analyze the umbilical venous catheter (UVC), the probe was placed vertically on the sternum with rotation to the right shoulder (to enable the catheter's in-plane analysis); for umbilical arterial catheter (UAC) visualization, the probe was placed in the midsagittal plane, lower than for UVC screening, with the upper part of the probe on the sternum and the lower part on the celiac plexus (Figure 1). A successful tip identification was defined as an image of a sharply contrasting double-contoured echoic structure.

Ultrasound screening consisted of:

1. assessing the catheter tip, location, movability, and thrombi-free surface and
2. analyzing the catheter trajectory: presence of surface thrombi and potential flow disruption (analyzed flow for the UVC: left and right portal vein, disruption of foramen ovale valve; for the UAC: renal arteries, superior mesenteric artery, celiac trunk).

### Outcome Variables

The outcome variables were defined as:

1. **catheter dislocation**. The proper catheter's position was defined as outside of the liver (distal part of ductus venosus), inferior vena cava or right atrium lumen for UVC and thoracic aorta, above the diaphragm for UAC. A position of the catheter other than those listed above was considered incorrect.
2. **catheter-related thrombosis** defined as any UAC thrombus or clinically significant UVC thrombus (causing altered portal vein flow, liver parenchymal irritation and thrombosis, risk of detachment into the heart chambers).

Based on follow-up examinations performed during hospitalization, the following actions were undertaken:

1. **leaving the catheter in place** for further monitoring;
2. **withdrawal to a proper position** within the first day of catheterization if the location was too deep (UAC in the upper part of the thorax aorta; UVC in the left atrium through the foramen ovale);
3. **removal of the catheter** if the location was too shallow (UAC below the renal arteries; UVC in the liver, i.e., ductus venosus, left branch of the portal vein or umbilical vein) or catheter-related thrombosis were detected or a sudden lack of catheter movement and adherence to the walls of the vessel was observed (as the concentrated hyperosmolar solution administered may irritate the vessel, cause thrombosis or increase vessel permeability, leading to heart tamponade).

### Statistical Analysis

Central tendency and variability measures were used to describe studied population. The birth weight and gestational age were presented as a median and range, other quantitative variables as mean and standard deviation (SD) values. The qualitative variables were presented as number and percentage (*n*/%).

The research was approved by Ethics Committee (no. 122.6120.287.2015 of the Jagiellonian University Bioethics Committee). All parents signed informed consent form before study procedures. The study was supported by National Science Center, Poland, as a part of project no. 2016/21/N/NZ5/01442. Deidentified participant data are available upon reasonable request from the first author.

## Results

**Figure 2:**
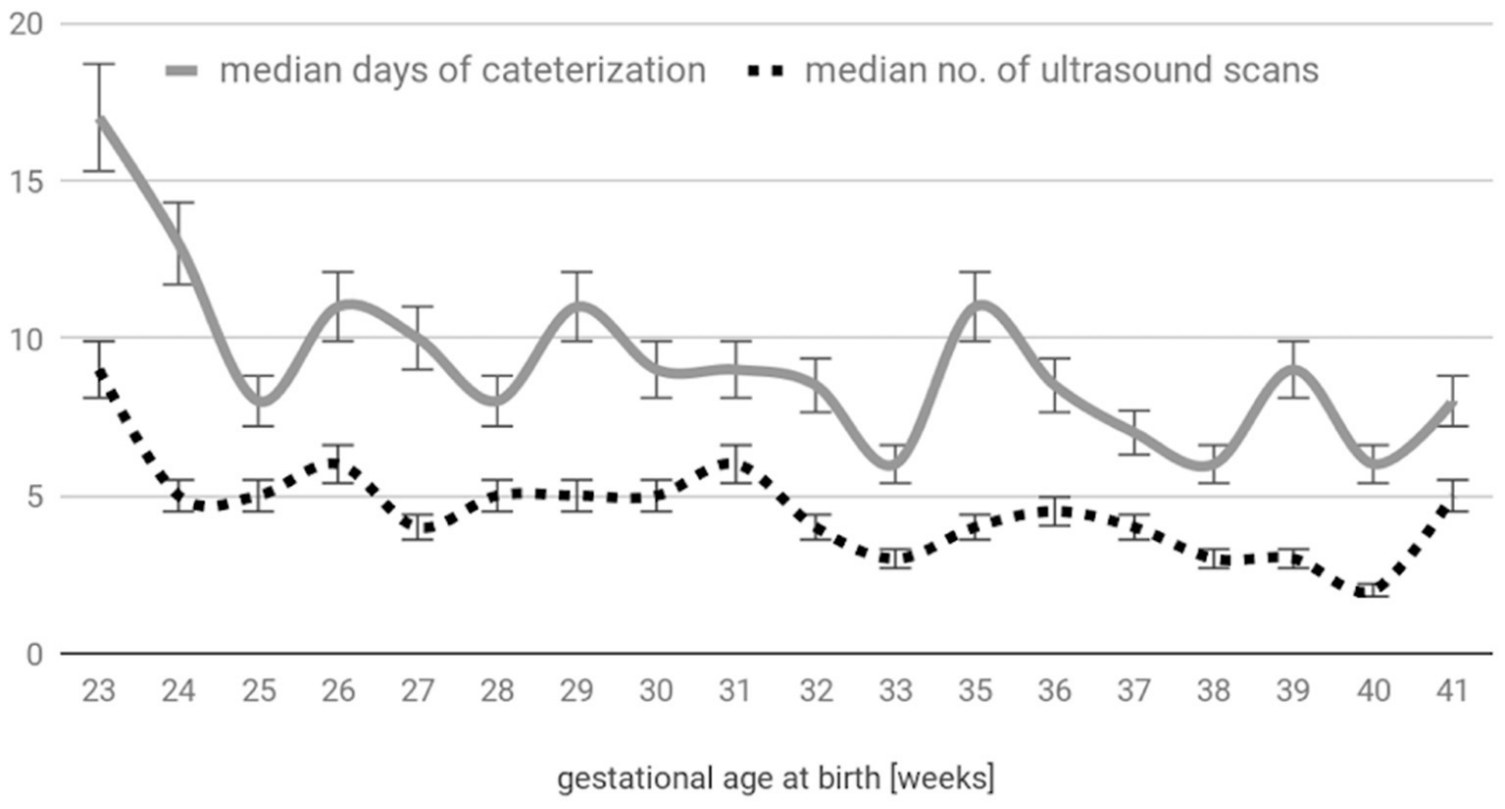
Distribution of the catheterization time and number of ultrasound scans with respect to gestational age at birth.

**Figure 3:**
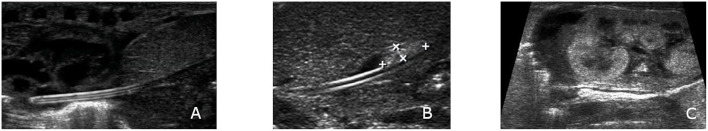
Ultrasound abnormalities: **(A)** UVC in a too-deep position (left atrium), **(B)** catheter thrombus in the LPV, and **(C)** thrombi cast in the infrarenal aorta post UAC removal.

A total of 129 patients (74 male, 55 female) were enrolled in the study; 194 umbilical catheters (119 venous and 75 arterial) were prospectively observed. The main reason for umbilical catheterization was prematurity (82%, *n* = 106; including 39 extremely premature patients); other causes were therapeutic hypothermia monitoring (13%, *n* = 17), meconium aspiration syndrome (*n* = 3), congenital disorders (*n* = 2), and severe anemia (*n* = 1). The patients' gestational ages ranged from 23 to 41 weeks (median 29 weeks), and their birth weights ranged from 460 to 3,620 g (median 1,200 g). A total of 954 examinations were performed (Figure 2). In only 1.5% (14/954) of cases, the umbilical catheter was not visible, and this situation occurred only in the venous group. The dwell time ranged from 2 to 35 days (the latter for an extremely edematous preemie who was unable to be decannulated); the mean catheterization time was 9 days for UAC and 11 days for UVC. The complications are compared in Table 1 and Figure 3.

**Table 1:** Comparison of catheterization complications.

**Outcome**	**UAC (*n* = 75)**	**UVC (*n* = 119)**
DISLOCATION	17 (23%)	81 (68%)
	To a crucial place: 0	To a crucial place: 63 (53%)
Clinical decision: intervention	0	54 (86%)
THROMBI during catheterization	6 (8%)	41 (34,5%)
Clinical decision: catheter removal	6 (100%)	23 (56%)
THROMBI visible only post catheterization	3 (4%)	1 (0.8%)

### Catheters Dislocation

Displacement of catheter throughout the whole dwell time was observed in 81/119 (68%) of UVCs and 17/75 (23%) of UACs. UVCs had a tendency to retract out of the patient with time (43/81—53%), while the UAC in 76% (13/17) of displaced cases was found in a deeper location than at first. In the case of UACs, the movement process was not significant enough to make a intervention necessary, whereas for UVCs, where even a few millimeters of dislocation may lead to a completely different hemodynamic situation, an intervention was needed in 54/63 (86%) of cases. The most common abnormal UVC location was the left atrium (54%, 34/63), from which the catheter was usually withdraw within the first hours of catheterization; 36.5% (23/63) of dislocated catheters were found in the middle of the ductus venosus, where the blood flow was residual. With the increased ultrasonographic monitoring-intervention approach, 77% (58/75) of UACs and 78% (93/119) of UVCs had a proper final location of the catheter tip: the supradiaphragmatic location for UACs and the distal part of the ductus venosus (16%, 19/119), inferior vena cava (19%, 23/119) or right atrium (43%, 51/119) for UVCs.

### Thrombosis

**Figure 4:**
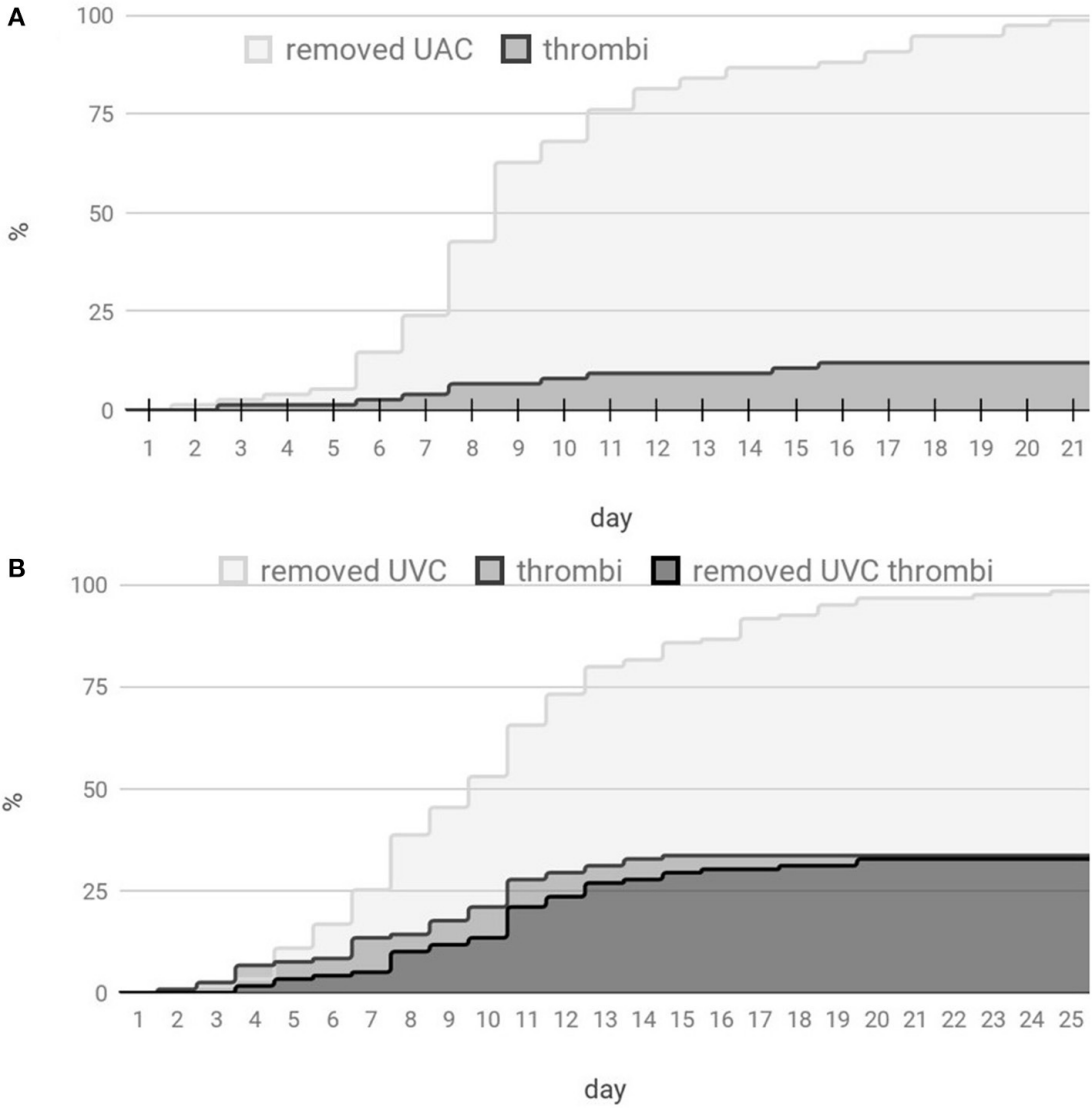
Distribution of the umbilical catheter count and thrombotic events with respect to the catheterization time. **(A)** Distribution for UACs and **(B)** distribution for UVCs.

Thrombotic complications during catheter dwell time observed in 41/119 (34.5%) of UVCs and (9/75) 12% of UACs (the time distribution is shown in Figure 4). Interestingly, three of UAC-associated thrombi were visible only after catheter removal (compared with 1 UVC-associated thrombus out of 119 UVCs), and almost all UAC related thrombi (8 out of 9) had left a postremoval sheath in the aorta. UAC thrombi had a tendency to locate near the aortic branches (mainly renal), whereas more than half of UVC thrombi were present in the ductus venosus region. During the prospective study, we observed that the majority of patients developed a surface-thrombotic/mossy catheter within the left portal vein—a phenomenon that did not affect the portal vein hemodynamics and was common (63% of UVC; 58/92); we did not consider this finding clinically significant. However, 27% (11/41) of the analyzed UVC thrombi were present in a crucial location, potentially leading to embolic events (distal part of the DV 5/11 (45.5%), right atrium 5/11 (45.5%) or disrupting the portal circulation (RPV, 1/11—9%). No UAC thrombi were located at the tip of the catheter; in contrast, tip thrombi were present in 13/41 (32%) of the UVC patients. Due to the crucial location, all UAC thrombi led to catheter removal. 44% (18/41) of observed UVC thrombi were left in place accompanied by intensified ultrasound monitoring. Moreover, 2/3 of catheters that were improperly located in the liver developed thrombi.

Based on the ultrasound image, the following action was undertaken: (1) none [69/75 (92%) of UACs and 58/119 (49%) of UVCs]; (2) withdrawal shortly after catheterization [*n* = 1 UAC; 40/119 (34%) of UVCs]; and (3) removal of the catheter [6/75 (8%) of UACs, 29/119 (24%) of UVCs]. In total, 51% (61/119) of UVC patients and 8% (6/75) of UAC patients required an intervention due to the obtained catheter image. The main cause of UVC removal was an incorrect (too shallow) position together with thrombotic complications [55% (16/29)], and thrombus alone was a cause of 24% (7/29) of UVC removals. The main cause of UAC removal was the lack of catheter movement probably due to ongoing thrombus formation. This may lead to other complications, such as injury to the aorta. Direct injury to the abdominal aorta—dissection due to an improper (too large) catheter size was observed in 1 of 75 patients.

## Discussion

This study prospectively analyzes ultrasound techniques for assessing complications of umbilical catheterization. To date, there is a lack of similar studies, and the following problem is emerging: 1 in 10 children is born premature (4), often requiring intensive care; moreover, the complications of catheterization, which are difficult to detect, can lead to potentially deadly conditions.

The strength of our study is a large number of umbilical catheters thoroughly evaluated by repeated ultrasound. Therefore, the risk of certain complications can be quantified more accurately than in previous reports. As in the majority of studies, the design of our research has limitations—it is mainly a single center descriptive study without sophisticated statistics. Furthermore, many factors can influence the rate of catheter-related complications—for example methods of catheter insertion and fixation, rules of catheter tip positioning, clinical decisions about the dwell time. Most of these factors may be dependent on the habits specific for the NICU where the research was conducted. Thus, the results observed in our center may differ from other units and may not be generalizable. Nonetheless, this study can help quantifying risks and determining the best catheter dwell time for neonatal care.

### Imaging

In our study, we performed nearly 1,000 ultrasound examinations of patients and experienced imaging problems in only 1.5% of the cases, never in the UAC group. The obtained images facilitated clinical decision-making, and we believe that is why we did not have any patients with clinical presentations of thrombosis, cardiac tamponades, arrhythmias, or other complications described in the literature.

The current standard method for evaluating the position of central lines is still conventional radiography performed once after insertion and again in cases with complications. There are also no recommendations suggesting the use of ultrasound simultaneously with catheter insertion; the proper position of the catheter is assessed in very vague ways, for example with the Shukla equation based on the child's birth weight. This equation was derived from regression equations of a very small group of 40 patients, which makes it statistically insignificant (5). The other formulas used for catheter positioning are Wright modification of the Shukla equation for ELBW infants (6) or the Dunn method [a nomogram based on the shoulder-to-umbilicus length, assessed on postmortem measurements of 50 infants (7)]. Research has shown that only 14 of 101 professionals use this method correctly (8). As the anatomical conditions of each child vary, there is no hope for obtaining a universal formula for proper catheter depth; rather, an *in vivo* monitoring system is required. Within a standard procedure, after insertion, the catheter is verified with an anteroposterior thoracic-abdominal X-ray image: the optimal UVC position (between the RA and IVC) (9) is described as a visible location of the catheter tip between the 8th and 10th thoracic vertebrae (10); research has shown that the level of the IVC/RA junction can vary widely from the 6th to 11th vertebrae (11). Multiple studies have questioned the ability of a radiograph to accurately evaluate the tip of an umbilical catheter (11–17). In a retrospective study by Harabor and Soraisham (17), 44% of ELBW infants had the UVC inserted too deeply despite X-ray showing a proper position. The X-ray image can be affected by a child's movement, an overlying temperature probe, congenital defects or a severe condition of the patient. Moreover, it is a harmful technique due to radiation and is especially dangerous for an undeveloped premature body. Data based on radiographs in NICU patients with NEC showed a relative risk of lifetime cancer mortality 4–20 × higher than that of baseline controls (18). Recently, the Society for Pediatric Radiology together with the American Society for Radiologic Technologists raised a campaign called “Image Gently,” whose role is to raise awareness of radiation risks in children and to recommend the use of alternative imaging modalities if possible^1^. In 1982, real-time ultrasound imaging of umbilical catheters was first used by Oppenheimer (12); since then, it still has not become popular enough for routine use. Thanks to the vascular lumen and well-defined walls of the catheter, as well as the small patient size with uncalcified bones, ultrasound imaging is easy, quick, harmless, and available at the bedside. Studies have shown that ultrasound imaging of umbilical catheters can detect abnormal positioning even when the X-ray indicated a correct position (11, 19). Moreover, ultrasound imaging can provide more information than a regular X-ray, including catheter movement, thrombotic complications or impaired flow, and can be a part of the whole general ultrasound assessment of such infants, which is crucial in neonatal critical care patients (who may exhibit patent ductus arteriosus, intracranial hemorrhages, development of necrotizing enterocolitis and other serious conditions).

### Proper Location Upon Insertion and Within Time

In our study, 43% of patients had a final location of the UVC tip within the right atrium, without any complications, which in our opinion confirms the safety of the described location while a proper monitoring protocol is implemented. However, recently, there has been a lot of controversy in the medical literature, regarding the accurate location of both UVC and UAC, despite the imaging technique.

Locating an umbilical arterial catheter directly in the aorta is a unique situation seen nowhere else in the medical setting. Cochrane metaanalysis (20) revealed that high (supradiaphragmatic) UAC position was associated with lower vascular complication rate than the low (infradiaphragmatic) location.

More questions arise when analyzing the proper position of the venous catheter; en route, it crosses anatomical sites with various hemodynamic conditions. By crossing the liver, the venous catheter may easily enter straight into the heart, and this used to be the major concern regarding UVC catheterization and is still, as we believe, a common misconception regarding UVC catheterization; from our observations, most of the complications occur within the liver. A freshly inserted catheter is elastic, easily bending in response to any obstacles on its way, as it is able to curl. We did not observe any cardiac perforation caused by catheter insertion during study period, however, we did observe injury of the delicate hepatic parenchyma leading to major hematomas caused by the adverse catheterization. As we proved in our previous research (21), catheters calcify with time and change their properties; an old, stiff UVC can perforate the heart, which is why intense catheter monitoring is required, especially given the fact that we demonstrated common time-dependent catheter dislocation. Moreover, it is important to realize the damage that can be done by the fluids administered via a catheter; a UVC is mostly used for parenteral nutrition (amino acids, lipids, carbohydrates, ions, and vitamins solution), which due to its high osmolarity can easily irritate the surrounding tissues causing inflammation, inducing necrosis and stimulating thrombosis; it is crucial to place the tip of the catheter in a way in which it will be intensively flushed by the surrounding blood flow. Average cardiac output is 300 ml/min/kg, which contributes ~600 ml/min flow in a heart chamber of a 2 kg child; the flow of a parenteral nutrition is ~150 ml/kg/24 h, which is 3,000 times slower than the blood flowing around the catheter when it is in the atrium. In contrast, the flow in the ductus venosus is residual, as the vessel is useless postnatally; the portal vein flow is ~50 ml/kg/min, as there is a double capillary net before and after the vein, which significantly slows the flow. Moreover, we administered parenteral nutrition directly to the portal system, bypassing the systemic circulation. There are data supporting the notion of the harmful effect of catheters placed in the portal system, causing necrosis, thrombosis, vasospasm (22), NEC and SIP (23, 24). Our observations based on years of ultrasound catheter monitoring are relevant: a shallow position of the UVC (within the liver, in the DV) quickly leads to liver irritation and thrombosis. There has been a conflict throughout the years between the common radiologist recommendation of the RA position (25) and neonatologist disapproval of the heart location (22, 26, 27). Based on research and years of experience, we suggest that having the tip of UVC within the right atrium may be safe, as long as we implement a regular ultrasound monitoring protocol, excluding prolonged adherence of the catheter tip to the heart wall. In the latter situation, the tip is no longer surrounded by blood, and the administered hyperosmolar fluid can cause wall erosion, which in worst case scenarios will lead to heart tamponade (vs. legendary perforation of the heart wall *per se*). We should also be aware of possible migration of the catheter via a still patent foramen ovale to the left atrium; this is a dangerous location not only regarding thrombotic events but also for lipid administration, which can lead to embolism within the systemic circulation.

### Displacement With Time

There are no recommendations for monitoring inserted catheters, despite multiple reports of umbilical catheter migrating into an unsafe position over time. Umbilical catheter migrate with time, independent of the catheter fastening technique (28). It is especially crucial for UVCs, where millimeters of displacement lead to a completely different hemodynamic situation with further consequences of hyperosmolar fluid administration. UVC tip migration was examined by Franta et al. (29); 62% of their patients had a malpositioned catheter, which went deeper into the LA (38/40). Greenberg et al. (13) showed that 56% of their UVCs needed to be repositioned, thus requiring more than one radiograph. Indirect proofs of catheter displacement (too deep) include reports of cardiac arrhythmias that resolve after umbilical catheter removal/withdrawal (30, 31); catheters with too deep positions can irritate the electric conduction system.

Other findings along with our results have indicated that migration usually occurs within the first 24–48 h of catheterization in ~¼ of all catheters, usually leading to a deeper location (28, 29, 32). This may be due to drying and shortening of the umbilical stump (28), abdominal distension when introducing enteral feeding, breathing support (nCPAP) compressing the liver, physiological weight loss and other factors affecting the size and shape of the liver. Although the most common abnormal position of the UVC was the LA as described above, we also noticed significant catheter displacement with time; more than half of catheters were found in a shallower place than was originally intended, leading to potential thrombotic events in the liver.

### Thrombosis

In our study, 50% of UAC-associated thrombi were detected only after catheter removal. That is why the authors believe that in the case of UAC even intense monitoring is not as efficient in screening for complications as in UVC. Furthermore, our observation of the numerous thrombi in the renal region indicates the importance of screening the renal arteries in patients with UAC.

Interestingly, we frequently observed thrombosis of the catheter on its way within the LPV, in most cases not affecting liver flow. However, intense monitoring is needed, as such thrombi might spread to the RPV, a branch that is crucial for liver flow, and ¾ of the blood supply to the liver comes from the portal flow (and 50% oxygenation).

Catheters are the main cause of thrombosis in the neonatal population and are responsible for 90% of cases (33). It was proven repeatedly that the catheter, as a foreign body, has a damaging effect on the endothelium, inducing inflammation and thrombosis. Most thrombi are asymptomatic, and their presence has been showed on autopsy; in a study by Tyson et al. (34), 59% of UAC infants had severe catheter-related thrombosis on autopsy. Rates are lower when the screening is performed *in vivo*; ¼ of UACs by aortography (35–37) and 30% of UVCs (38). Surprisingly, most of the studies proved that the risk of thrombosis is not directly related to the catheterization duration time (34–36), which was also observed in our study; some thrombotic events were observed quickly within the first days of catheterization. We are especially aware of aortic thrombosis, which has a high mortality because the vessel is crucial for organ flow and cannot be treated surgically (due to its small diameter) or pharmacologically (fibrinolysis is contraindicated in the preterm population). The timing of aortic catheterization should be as short as possible, and there is no need to maintain the UAC if the neonate does not require continuous blood pressure monitoring or frequent arterial blood sampling.

Ultrasound monitoring in intensive care units has become the stethoscope of the 21st century, helping detect abnormalities before their clinical manifestation. It does not require a high level of technical expertise, is not time consuming, and can be effective even after basic training (39). Moreover, among all patients, neonates are the easiest to evaluate with ultrasound thanks to their uncalcified bone structure, low fat tissue and high hydration status. We therefore emphasize the need for point-of-care training and accreditation to use sonography as the primary evaluation technique for umbilical catheters.

## Conclusions

With this paper, we arrive at the following conclusions:

1. US imaging allows a precise assessment of the umbilical catheters' surface and tip position (only 1.5% of catheters are not visible upon examination).
2. The most common UC complication is catheter dislocation (68% of UVCs and 23% of UACs).
3. UC thrombosis rarely occurs within the first 7 days of catheterization.
4. UVCs are prone to induce catheter-related thrombosis (1/3 of cases), yet not all of them require removal if systematic monitoring is implemented.
5. UAC thrombi, although rare, leave a thrombotic sheath in the aorta; some of them can be detected only after catheter removal.
6. Bedside ultrasound imaging of catheters supports the decision-making process related to the catheterization duration, shortening the time if abnormalities are detected and allowing a safer prolonged UVC stay when an alternative central line cannot be assessed.

## Data Availability Statement

The raw data supporting the conclusions of this article will be made available by the authors, without undue reservation.

## Ethics Statement

The studies involving human participants were reviewed and approved by Jagiellonian University Bioethics Committee approval no. 122.6120.287.2015. Written informed consent to participate in this study was provided by the participants' legal guardian/next of kin.

## Author Contributions

PKw, AS, and PKr made substantial contributions to the design of the study. AS and AD collected, analyzed, and interpreted the research data and they were major contributors in drafting the initial version of the manuscript. PKw and PKr reviewed and revised the manuscript. All authors read and approved the final manuscript and agree to be accountable for all aspects of the work.

## Conflict of Interest

The authors declare that the research was conducted in the absence of any commercial or financial relationships that could be construed as a potential conflict of interest.

## Footnotes

^1^https://www.imagegently.org
